# Otolaryngologists and the Early Diagnosis of Mucopolysaccharidoses: A Cross-Sectional Study

**DOI:** 10.3390/diagnostics9040187

**Published:** 2019-11-13

**Authors:** Danielle de Araujo Torres, Anneliese Lopes Barth, Mariana Pires de Mello Valente, Paulo Pires de Mello, Dafne Dain Gandelman Horovitz

**Affiliations:** Medical Genetics Department, National Institute of Women, Children, and Adolescents Health Fernandes Figueira, Oswaldo Cruz Foundation, Av Rui Barbosa 716, Rio de Janeiro 22250-020, Brazil; annebarth@hotmail.com (A.L.B.); paulopmello@ibest.com.br (P.P.d.M.)

**Keywords:** mucopolysaccharidoses, otolaryngology, early diagnosis, respiratory tract

## Abstract

Mucopolysaccharidoses (MPS) are a group of inborn errors of metabolism with an aggressive and usually fatal course. Therefore, early treatment is essential because the involvement of head and neck structures is almost always present in MPS. Our study aimed to retrospectively assess—via a chart review and a survey of caregivers—the history of ear, nose and throat (ENT) symptoms, the number of otolaryngology visits prior to diagnosis, and whether otolaryngologists diagnosed the disease in a cohort of MPS patients followed at an academic medical center. Twenty-three patients were evaluated. Age at diagnosis ranged from 0.2 to 33.0 years (median, 3.2 years). Prior to being diagnosed with MPS, 20/23 (87%) patients presented with at least one episode of otalgia, airway disorder, sleep disturbance, speech delay or suspected hearing loss. One patient had an adenotonsillectomy with paracentesis of tympanic membranes. Ten of the 23 patients (43%) were seen by an otolaryngologist before the diagnosis of MPS, none of which had the disease suspected during these visits. Notwithstanding limitations, our results suggest that increased awareness of MPS among otolaryngologists may allow for earlier diagnosis and better management of these patients.

## 1. Introduction

Mucopolysaccharidoses (MPS) are a group of rare genetic disorders with an estimated incidence of 1.75–4.5 cases per 100,000 live births [1,2,3,4,5,6,7,8,9]. These inborn errors of metabolism are characterized by the deficiency of specific lysosomal enzymes, which causes glycosaminoglycan to accumulate in the connective tissue, ultimately resulting in variable degrees of multiple organ dysfunction [10].

Seven distinct clinical types and numerous subtypes of MPS have been identified, according to the specific enzyme defect and severity of symptoms. In all but milder cases, MPS are typically present in infancy or early childhood with a very aggressive and often fatal course, averaging a life expectancy of one or two decades if left untreated [11].

Although initial MPS symptoms are usually insidious and non-specific, the involvement of head and neck structures is an early and almost universal finding in MPS patients, which includes upper airway obstruction, recurrent otitis and sinusitis, adenotonsillar hypertrophy, hearing loss and coarse facies [12,13].

These observations underscore the importance of otolaryngologists for the prompt diagnosis and treatment of MPS patients [14,15], particularly those with MPS types I, II, IV-A and VI, for whom enzyme replacement therapy (ERT) can be an effective approach at disease onset, preventing or delaying somatic deterioration when irreversible organ damage is yet to occur [16,17].

Therefore, the purpose of this study was to assess the history of ear, nose and throat (ENT) symptoms and the number of otolaryngology visits prior to the diagnosis of MPS and to determine whether the otolaryngologist was able to detect the disease in a cohort of MPS patients followed at an academic medical center.

## 2. Materials and Methods

All patients with a confirmed MPS diagnosis by enzyme activity analysis who were being followed at the Medical Genetics department, National Institute of Women, Children and Adolescents Health (Rio de Janeiro, Brazil) in 2015, regardless of age or treatment status, were selected for a manual chart review. Since the clinical phenotype and natural history of MPS type III is so remarkably different from the other types [11], being a predominantly neurodegenerative disease, MPS III patients were excluded from the analysis.

Medical records were used to gather patient and disease characteristics, and previous and current therapies that were used. The primary caregivers of the individuals with MPS were asked to complete a survey consisting of 12 questions designed to retrospectively evaluate the history of ENT symptoms and the number of otolaryngology consultations before the diagnosis of MPS, and to ascertain if the otolaryngologist was the diagnosing physician. Descriptive statistics were calculated for all responses.

Informed consent was obtained for all individuals. The study (registered under the number CAAE: 0074.0.008.000-11) was approved in December 12, 2011 by the local ethics committee on human research (Comitê de Ética em Pesquisa- Instituto Fernandes Figueira) and followed the ethical principles of the Declaration of Helsinki [18].

## 3. Results

Five patients with MPS I, seven with MPS II, one with MPS III, four with MPS IV-A, and seven with MPS VI were identified. One MPS II patient who was diagnosed prenatally and transplanted at age 2 months was excluded from the analysis. One MPS III patient was also excluded as per protocol, leaving a total of 23 evaluable patients. The median age at the time of the analysis was 10.6 years (range, 2.1–37.6 years). The median age at the time of the diagnosis of MPS was 3.2 years (range, 0.2–33 years). Fifteen patients were male.

According to their primary caregivers, 20/23 (87%) patients presented with at least one of the following ENT symptoms prior to the diagnosis of MPS: otalgia, airway disorders, snoring, respiratory distress at night, sleep apnea, speech delay, and suspected hearing loss (Table 1). Among the patients with otalgia, the median number of episodes was 2.5 (range 1–10).

One MPS II patient underwent an adenotonsillectomy with paracentesis of tympanic membranes at the age of 2.9 years before the MPS diagnosis was considered. Five other patients had other surgical procedures before the MPS diagnosis—four were submitted to umbilical herniorrhaphy, and one had a cardiac valvuloplasty. The median interval between surgery to diagnosis of MPS was 28.2 months (Table 2).

Unfortunately, due to various difficulties in our public health system, many still have not had access to ENT surgery. Overall, in our sample, four patients had an adenotonsillectomy (1 MPS I, 1 MPS II and 2 MPS VI, one before MPS diagnosis), despite surgery being recommended in 20/23 cases.

Adenotonsillar hypertrophy was observed in all MPS subtypes, as well as middle-ear effusion, upper airway obstruction, and supraglottic mucosa redundancy. Adenotonsillar hypertrophy was only absent in a 2-year-old MPS I patient who underwent hematopoietic stem cell transplantation (HSCT), in a 7-year-old MPS VI patient, and in a 9-year-old more attenuated MPS VI patient.

Tracheal deformities were more pronounced in MPS IV and VI patients despite also being present in other groups. All our patients underwent endoscopic evaluation of upper and lower airways. Significant tracheal deformity was observed in MPS IV-A and VI patients, with more specific findings when compared to the other MPS types. Severe tracheal obstruction occurred in a MPS II 10-year-old boy, who had a tracheostomy and needed a longer cannula; he ultimately died from respiratory complications. 

With regards to clinical history and previous evaluations registered in the survey, ten of the 23 patients (43%) were seen by an otolaryngologist before the diagnosis of MPS. The median number of ENT consultations was 2.5 (range, 1–10). All of these 10 patients developed sleep disturbances at some point before the diagnosis. All but three had at least one episode of otalgia prior to being diagnosed with MPS. None of these patients had MPS diagnosed or even suspected by the otolaryngologist.

Two patients (one MPS I and one MPS II) underwent hematopoietic stem cell transplantation before 1 year of age and were not on ERT. Both presented normal airways.

A 16-year-old female patient with MPS IV-A had not yet started ERT at the time of the analysis. The remaining 20 patients were receiving ERT according to the current guidelines. ERT has significantly reduced both the number and severity of upper airway infections in all cases, although structural abnormalities, such as adenotonsillar hypertrophy and tracheal deformities, were still present and progressive despite the recombinant enzyme treatment. 

There is a correlation between height and the severity of ENT symptoms. In general, the most severely affected are shorter, while the attenuated are taller. This is also related to the clinical presentation: the attenuated patients tend to be taller. In Brazil, most of our patients have the severe form, therefore, they tend to be shorter and presenting many ENT symptoms, combined with other co-morbidities

## 4. Discussion

MPS are serious and progressive medical conditions that can go undetected for many years. During this pre-diagnosis period, MPS patients usually visit the emergency room multiple times and undergo consultations with different medical specialists—including otolaryngologists, as our study confirms. Unfortunately, a delay in diagnosis can have a profoundly negative impact on treatment outcomes, as both ERT and hematopoietic cell transplantation are most effective when carried out as early as possible in patients without advanced organ damage [19]. Therefore, early referral to a metabolic specialist or to a clinical geneticist is of paramount importance for MPS patients.

**Figure 1:**
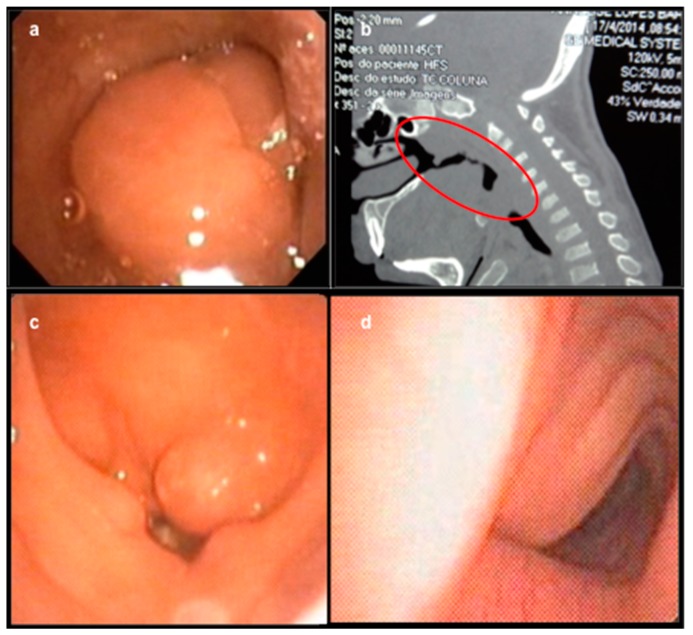
(**a**) Adenoid hypertrophy in a 12-year-old MPS VI patient on ERT since age 5. (**b**) Upper airway obstruction in a 9-year-old MPS VI patient on ERT since age 3. (**c**) Redundant supraglottic mucosa in a 15-year-old MPS VI patient on ERT since age 7. (**d**) Tracheal deformity in an 11-year-old MPS IV-A patient on ERT since age 8.

Airway diseases are among the first manifestations of MPS (Figure 1a–d). Recurrent otitis and sinusitis, mucoid or serous middle ear effusions, adenotonsillar hypertrophy, upper respiratory tract viral infections, upper airway obstruction, and sleep disorders are common findings in MPS, although very prevalent in the general pediatric population as well [20,21]. However, otolaryngologists should pay close attention to younger-than-usual patients with more severe manifestations of ENT disorders [14]. The coexistence of other clinical signs, such as hernia, skeletal abnormalities, stiffness and joint contractures, clawed and short fingers, corneal clouding and vision loss, as well as hepatosplenomegaly, should also raise suspicion of MPS [22].

Coarse faces are another hallmark of MPS, which includes macrocephaly with frontal prominence, hypertelorism, flat nasal bridge, flared nostrils, thickened lips, short mandibular rami, gingival hyperplasia, hypoplastic peg-shaped teeth with delayed eruption, and macroglossia [23]. The presence of these signs should also prompt referral to a specialist, especially if combined with ENT manifestations.

In addition to the poor prognosis associated with delayed diagnosis, the fact that many MPS patients undergo surgical procedures before being diagnosed with the disease—as seen in our cohort—is of great concern [15]. Tonsil and adenoid surgery—one of the most performed surgical procedures in MPS patients—may be considerably more difficult due to the musculoskeletal manifestations of the disease in the head and neck region [24,25]. MPS patients may also present with atlantoaxial instability and/or cervical cord compression, thus contraindicating the hyperextension of the neck [26,27].

Furthermore, the anesthetic risk is extremely high in these patients, due to deformities of the larynx, trachea and the entire lower respiratory tree. Intubation is often only possible with the aid of a flexible pediatric bronchofibroscope [28]. Obstructive sleep apnea syndrome (OSAS) is associated with an increased risk of cardiorespiratory complications, while coexistent cardiac valve disease may also require antibiotic prophylaxis to prevent infectious endocarditis [29].

Our study has many limitations. The survey relied solely on caregivers’ recollections of events. It did not allow us to determine the point of the disease course when the patients were seen by otolaryngologists or whether other symptoms and signs that might suggest MPS were present at that time. Moreover, it was not possible to learn if, and which, additional tests were performed at the time of the otolaryngology evaluations.

## 5. Conclusions

The high prevalence of ENT complaints and the high number of otolaryngology consultations prior to the diagnosis of MPS, coupled with the fact that none of the otolaryngologists visited by this patient cohort raised a clinical suspicion of MPS indicate that efforts toward increasing awareness of MPS among these specialists have the potential to allow for earlier diagnosis and, hopefully, better management of MPS patients.

## Figures and Tables

**Table 1:** Ear, nose and throat (ENT) problems that arose prior to the diagnosis of Mucopolysaccharidoses (MPS), according to primary caregivers.

ENT Problems	MPS Patients (*n* = 23)
Otalgia, *n* (%)	10 (43)
Airway disorders, *n* (%)	13 (57)
Snoring, respiratory distress at night and/or sleep apnea, *n* (%)	15 (65)
Speech delay, *n* (%)	12 (52)
Suspected hearing loss, *n* (%)	9 (39)
At least one of the above, *n* (%)	20 (87)

**Table 2:** Surgical procedures performed before the diagnosis of MPS.

MPS Type	Surgical Procedure	Age at the Surgical Procedure (yrs.)	Age at the Diagnosis of MPS (yrs.)	Interval from Surgery to Diagnosis (mo.)
I	umbilical herniorrhaphy	3.0	7.5	54.0
II	umbilical herniorrhaphy	6.0	17.4	136.8
II	umbilical herniorrhaphy	30.0	33.0	36.0
II	adenotonsillectomy with paracentesis of tympanic membranes	2.9	3.9	12.0
VI	umbilical herniorrhaphy	0.8	2.5	20.4
VI	cardiac valvuloplasty	1.1	1.6	6.0

yrs: years; mo: months.

## Abbreviations

MPS	Mucopolysaccharidoses
ENT	Ear, nose and throat
ERT	Enzyme replacement therapy
